# Modifiable sleep-related risk factors in infant deaths in Cook County, Illinois

**DOI:** 10.1186/s40621-019-0203-1

**Published:** 2019-05-29

**Authors:** Anna Briker, Suzanne McLone, Maryann Mason, Nana Matoba, Karen Sheehan

**Affiliations:** 1 Feinberg School of Medicine, Northwestern University, Chicago, IL USA; 2 Injury Prevention & Research Center, Stanley Manne Children’s Research Institute, Ann and Robert H. Lurie Children’s Hospital of Chicago, Chicago, IL USA; 3 Division of Neonatology, Ann & Robert H. Lurie Children’s Hospital of Chicago, Chicago, IL USA

**Keywords:** Safe sleep, Infant deaths, Disparities

## Abstract

**Background:**

Each year, approximately 3500 infants in the United States die from sleep-related deaths. The number of sleep-related infant deaths has decreased overall since the 1990s, but disparities in sleep-related deaths persist among different populations. The purpose of this study was to determine the most common risk factors and locations in Cook County, Illinois for sleep-related deaths in infants under 6 months of age.

**Methods:**

We conducted a retrospective study among infants less than 6 months of age who died in Cook County, Illinois in 2015 and 2016, in which the manner of death was of undetermined intent with at least one modifiable sleeping risk factor present, as reported by the medical examiner. Data were obtained from the Illinois Violent Death Reporting System (IVDRS), a state-based, anonymous, surveillance system. County trends and circumstances of the deaths were also evaluated. Frequencies, percentages, and Chi-square analysis were used to describe and characterize these deaths.

**Results:**

In Cook County in 2015 and 2016, 116 infants less than 6 months of age died where the manner of death was classified as undetermined intent. The median age of death was 2 months. Of these deaths, 63 (54.3%) of the infants were boys. African-American and Hispanic infants comprised 71 (65.7%) and 23 (21.3%) of the deaths, respectively. In 84 (72.4%) of the cases, at least one known sleeping risk factor was present and 56 (66.7%) of the infants who died with a known sleeping risk factor were co-sleeping. Notably, 33 (29.7%) of the deaths in Cook County were clustered within six zip codes.

**Conclusions:**

The majority of infants who died unexpectedly in Cook County in 2015 and 2016 did so in the presence of sleeping risk factors, with co-sleeping being the most common. African-American infants, infants under 2 months of age, and several geographical areas within Chicago appear to be at increased risk. Interventions to target these preventable causes in the populations at increased risk should be instituted to prevent future deaths.

## Background

Each year approximately 3500 infants in the United States die from sleep-related deaths (Sudden unexpected infant Death and sudden infant Death syndrome: CDC/NCHS, 1990–2016). In 1994, the National Institute of Child Health and Human Development (NICHD) began a “Safe to Sleep” educational campaign to address the pressing problem of sleep-related infant deaths. Although rates have decreased since the 1990s, the rate of decline in infant deaths has stagnated since 2000 (Bombard et al., 2018; Parks et al., 2017; Carlin & Moon, 2017).

Disparities in infant mortality rates and rates of sudden unexpected infant death (SUID) among different ethnic/racial groups within the United States have long been observed (Singh & Yu, 1995). In 2016, the SUID rate of non-Hispanic black infants was 177.3 deaths per 100,000 live births, and the SUID rate of American Indian/Alaska Native infants was 196.9 deaths per 100,000 live births. In comparison, the SUID rate of non-Hispanic white infants was 84.5 deaths per 100,000 live births and that of Hispanic infants was 51.7 deaths per 100,000 live births (Period Linked Birth/Infant Death Data [Internet], 2016).

Studies have identified behaviors associated with sleep-related infant deaths (Sauber-Schatz et al., 2015; Hauck et al., 2003). In 2016, the American Academy of Pediatrics (AAP) released a policy statement with key recommendations for safe sleep (Moon, 2016). Safe sleep practices, such as placing infants supine and alone on a firm sleep surface free of other objects and having infants share a room (but not a bed) with parent(s), varies widely. Trends in practices have been associated with factors such as geography, maternal age and education level and racial/ethnic group (Bombard et al., 2018; Parks et al., 2017). Identifying high risk groups and locations of sleep-related death enables the development of targeted interventions. Thus, with this study we aimed to determine the most common risk factors and locations in for sleep-related deaths in infants under 6 months of age in Cook County, Illinois.

## Methods

Data were obtained from the Illinois Violent Death Reporting System (IVDRS), which is part of the National Violent Death Reporting System (NVDRS). The IVDRS is housed at the Injury Prevention & Research Center at the Ann & Robert H. Lurie Children’s Hospital of Chicago and acts as a bona fide agent of the Illinois Department of Public Health (IDPH). This is a state-based surveillance program of the Centers for Disease Control and Prevention, National Center for Injury Prevention and Control (CDC Injury Center). The IVDRS collects data on deaths due to suicide, homicide, legal intervention, undetermined intent, and unintentional fatal firearm injury and is one of 42 sites (40 states and Puerto Rico and Washington, D.C.) participating in the NVDRS at the time of this analysis. Data are collected from multiple sources, including vital records, the coroner/medical examiner, law enforcement reports, and toxicology reports and are pooled together into an analyzable, anonymous database (National Violent Death Reporting System: CDC, 2018).

The cases in this study are infants under 6 months of age who died in Cook County, which contains the city of Chicago and suburbs, in which the manner of death was classified as undetermined intent. Manner of death of undetermined intent was chosen as the selection criterion because this is the category used for SUIDs in the state of Illinois. Infants who died with an unknown cause but the known presence of a modifiable sleeping risk factor (such as, for example, an infant who suffocated while sleeping with an adult) would be captured in this category. Age of less than 6 months was used as a selection criterion because this has been established as the highest risk time period for SUID (Parks et al., 2017; Shapiro-Mendoza et al., 2009; Colvin et al., 2014).

For all infants who met the inclusion criteria, an IVDRS team member reviewed the medical examiner file for any mention of a sleep-related risk factor discovered during the scene investigation. All available information about where and how the infant was sleeping was extracted and coded according to the AAP’s most recent policy statement on recommendations for safe sleep. Sleep-related risk factors are not routinely collected as part of IVDRS. Out of the AAP’s A-level recommendations, we were able to use data available in IVDRS to assess for three—(1) “using a firm sleep surface”, (2) “room-sharing with the infant on a separate sleep surface”, and (3) “keeping soft objects and loose bedding away from the infant’s sleep area” (SIDS and Other Sleep-Related Infant Deaths, 2016). We defined co-sleeping as sharing a sleep surface with another person, regardless of the location or identity of the other person such as parent or sibling. All cases had previously been included into IVDRS and abstracted according to CDC Injury Center protocols. In 100% of the cases, the medical examiner records in this sub-analysis were examined by a second IVDRS team member specifically to identify the presence of any sleeping risk factors.

We also collected three outcomes: cause of injury, immediate cause of death, and cause of death by underlying International Classification of Disease, tenth revision (ICD-10) codes for each decedent. While cause of injury, immediate cause of death, underlying cause of death ICD-10 code are closely related, these terms do not always align. Please see Table 1 for a summary of definitions of these vital records terms (Medical Examiners' and Coroners' Handbook on Death Registration and Fetal Death Reporting, 2003).

**Fig. 1:**
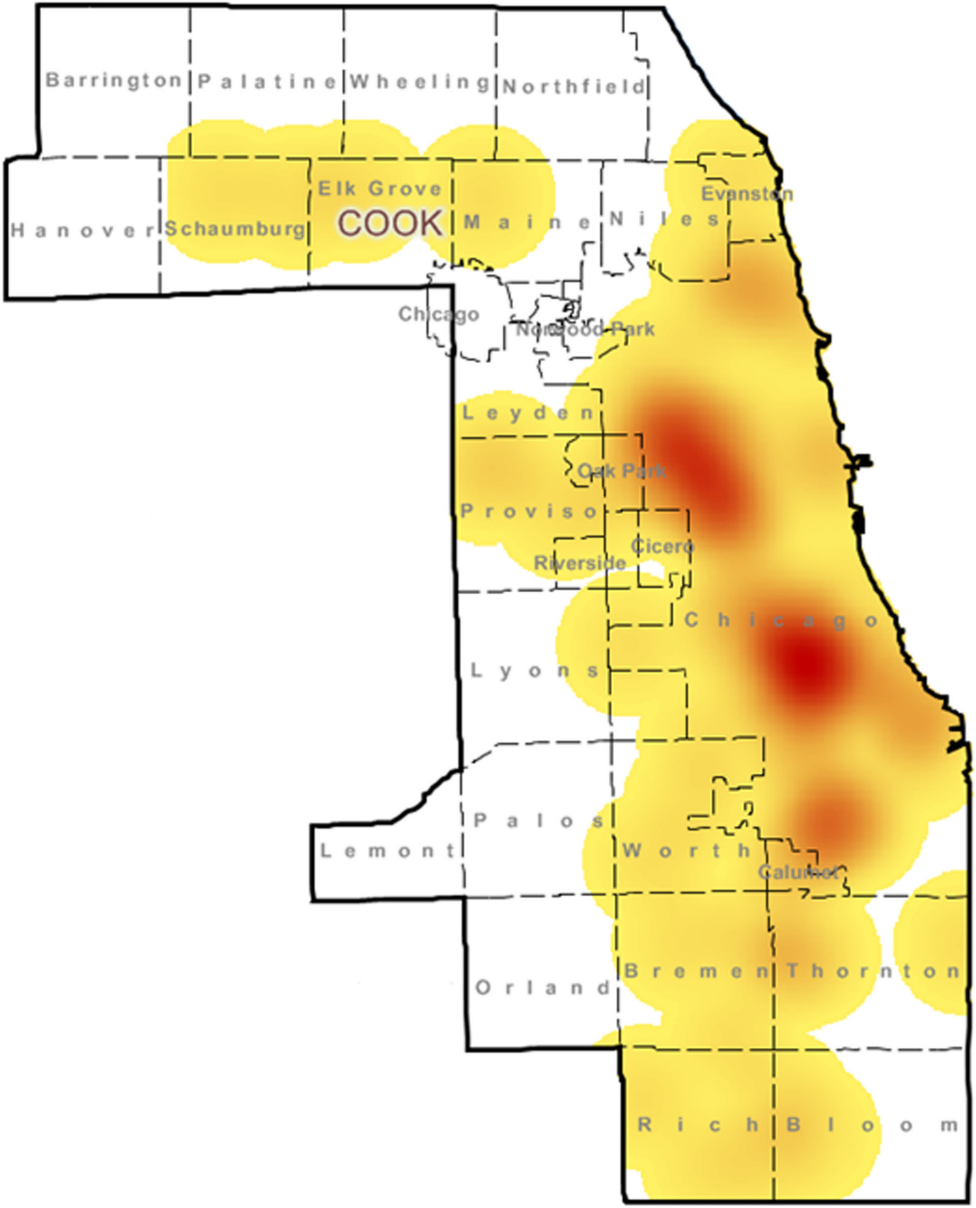
Depicts an incidence map of the infant deaths within Cook County in 2015 and 2016, with white indicating no death and red the highest incidence

**Table 1:** Summary of definitions

	Definition	Categorization
Manner of death	medical-legal: term listed on a death certificate	5 categories: natural, accident, homicide, suicide, undetermined intent
Cause of injury	mechanism by which decedent sustained (fatal) injury	brief description
Cause of death, by immediate cause	biological: “final disease or condition resulting in death”	2 categories: listed or could not be determined
Cause of death, by underlying code	biological: “disease or injury that initiated the events resulting in death”	International Classification of Disease, tenth edition (ICD-10) codes

Among the cases, race/ethnicity, sex, and age of the infants were identified. Zip code and census tract information were generated from the location of death of the decedent. Trends in location in the county and the circumstances of the deaths were also evaluated. In order to determine whether place of residence was the same as place of injury, if the place of injury was described as “home” or if the listed address was the same, we concluded that the place of injury and place of residence were the same.

Frequencies, percentages, and Chi-square analysis were used to describe and characterize distribution of these deaths by person and place to identify specific populations at increased risk. As part of IVDRS’s data-sharing agreement with IDPH to maintain confidentiality of the decedents, no data point that has a frequency of less than 10 may be reported; thus, several cells in the tables were suppressed. Missing data are inherent in vital statistics and in cases in which variables were not recorded within the database, this is stated. Density maps of census tracts where fatal injury occurred were created using Maptitude 2016 (Caliper Corporation, Newton, MA). Data were collected in software developed by the CDC for NVDRS; all analyses were performed using SPSS 22.0 (IBM, Chicago, Illinois). The study was deemed exempt from Institutional Review Board (IRB) review.

## Results

### Cause of death and injury

In 2015 and 2016, 116 infants under 6 months of age died in an undetermined manner in Cook County. This value represents 12.2% of the 953 infants under 12 months of age who died in Cook County in 2015 and 2016 (Infant Deaths and Infant Mortality Rates (IMR) by Resident County, Illinois Residents, 2015–2016). The number of infants specifically under 6 months of age who died in Cook County in these years is not made available by the IDPH. The causes of immediate death, of underlying death, and of injury are described in Table 2. Overall, the immediate cause of death was undetermined in over 90% of the cases. There were 11 different underlying causes of death, coded by ICD-10. In 104 (89.7%), “other ill-defined and unspecified causes of mortality” (ICD-10 code R99) or “sudden infant death syndrome” (ICD-10 code R95) were listed as underlying cause of death. Out of the 112 cases in which cause of injury was considered undetermined, sleep environment/external factors could not be ruled out as factors contributing to death in 98 (87.5%) cases. Autopsy was conducted in over 90% of the cases; in six cases, information about autopsies was not available.

**Table 2:** Characterization of manner of death, cause of death, and cause of injury (*N* = 116)

		Frequency	Percent
Manner of Death	Undetermined	116	100
Cause of death, by immediate cause^a^	Could not be determined	…	> 90
Cause of death, by underlying ICD-10 code	R99 or R95	104	89.7
	Other	12	10.3
Cause of Injury (*n* = 112)	Cannot exclude sleep environment/external factors contributing to death	98	87.5
	Undetermined or other	14	12.5
Autopsy conducted^a^	Yes	108	> 90

^a^some cells suppressed

### Demographics

The demographic characteristics of the study population are described in Table 3**.** Sixty (51.7%) deaths occurred in 2015. The median age of death was 2 months, with 49.1% occurring at less than 2 months of age. Sixty-three (54.3%) infants were boys. Seventy-one infants (65.7%) were African-American, and 23 (21.3%) were Hispanic. Information about race/ethnicity was not available for eight cases. Forty-nine (42.3%) deaths occurred on Saturday or Sunday. No trends were observed based on season or month of death. Of the 104 cases in which birthplace was known, over 90% of infants were born in Illinois, and 71 (68.3%) were born in Chicago. Over 90% of infants resided in Cook County. Of the 109 cases in which both place of residence and place of injury were known, 77 (70.6%) infants were at their listed place of residence when the injury occurred.

**Table 3:** Demographics (*N* = 116)

		Frequency	Percent
Year of death	2015	60	51.7
	2016	56	48.3
Age at death	< 2 months	57	49.1
	≥2 month -- < 4 months	37	31.9
	≥4 months -- < 6 months	22	19.0
Sex	Male	63	54.3
	Female	53	45.7
Race/Ethnicity (*n* = 108)	African American	71	65.7
	Hispanic/Latino	23	21.3
	Other	14	13.0
Day of week of death	Weekend (Sun. and Sat.)	49	42.2
	Weekday (Mon.-Fri.)	67	57.8
Season of death	Winter (Dec.-Feb.)	31	26.7
	Spring (March–May)	29	25.0
	Summer (June-Aug.)	24	20.7
	Autumn (Sep.-Nov.)	32	27.6
Birth city (*n* = 104)	Chicago	71	68.3
	Other	33	31.7
City of residence (*n* = 109)	Chicago	75	68.8
	Other	34	31.2
Injury location as compared to place of residence (*n* = 109)	Same injury location compared to residence	77	70.6
	Different injury location compared to residence	32	29.4

Mapping shows clustering of these deaths by geography (Fig. 1). Injury location was known for 111 cases, and these deaths occurred in 58 different zip codes throughout Cook County. Of these cases, 33 deaths (29.7%) occurred in just six zip codes and 57 deaths (51.4%) occurred in 14 zip codes (data not shown).

### Sleeping risk factors

The characteristics of sleeping risk factors are described in Table 4. Notably, only 13 (11.2%) cases lacked the presence of any reported sleeping risk factors. At least one sleeping risk factor was present in 84 (72.4%) cases. In 19 cases (16.4%), it could not be determined whether a sleeping risk factor was present. The most common sleeping risk factor was co-sleeping, defined as sharing a sleep surface with another person, regardless of the location or identity of the other person, such as parent or sibling. Well over half (56, or 66.7%) of the infants who died with a known sleeping risk factor were co-sleeping. Of the 28 infants with a known sleeping risk factor who were not co-sleeping, the two most common factors were sleeping in an adult bed and sleeping in a crib, bassinette, or playpen/portable crib that also contained other objects.

**Table 4:** Evaluation of sleeping risk factors (*N* = 116)

		Frequency	Percent
Presence of sleeping risk factor	Yes	84	72.4
	Could not be determined	19	16.4
	No	13	11.2
Among those with sleeping risk factor, presence of co-sleeping (*n* = 84)	Yes	56	66.7
	No	28	33.3

No statistically significant differences (*p* < .05) were observed in year of death, age at death, sex, race/ethnicity, or month of death between infants who died with and without sleeping risk factors (data not shown.) Additionally, there was no statistically significant difference in presence of sleeping risk factors between infants who died at their place of residence or at a different location than their place of residence.

## Discussion

In this study of infant deaths of an undetermined manner in Cook County, Illinois in 2015–2016, we found that the majority of deaths were associated with modifiable sleeping risk factors. The presence of sleeping risk factors, and in particular co-sleeping, suggests that many of these deaths may have been preventable. We identified other factors associated with increased incidence of sleep-related death, such as age younger than 2 months, minority race/ethnicity, and certain geographic regions. This study establishes methodology for characterizing SUID risk factors locally using NVDRS. Moreover, this data may facilitate the targeting of future prevention efforts to reduce infant mortality.

In our dataset, infants younger than 2 months comprised almost half of the deaths. This observation is consistent with other studies that have shown younger infants to be at higher risk. Shapiro-Mendoza et al. found that between 2002 and 2004, over 70% of cases of accidental suffocation or strangulation in bed occurred in infants under 3 months of age (Shapiro-Mendoza et al., 2009). Like other factors associated with SUID, the impact of age on risk is not yet fully elucidated. However, studies have shown that risk factors associated with death vary among different age groups, with co-sleeping being more commonly present among infants younger than 4 months of age who died suddenly (Colvin et al., 2014; Trachtenberg et al., 2012). In our dataset, almost two thirds of the deaths were African Americans. According to the American Community Survey 1-Year Estimates from the U.S. Census Bureau, African Americans comprised 31% and Hispanics comprised 36.7% of children under 5 years of age in Cook County in the same time period (U.S. Census Bureau [Internet], 2017). This observation is consistent with other studies that show racial disparities among infant mortality rates in the US in general and sleep-related infant death rates in particular (Parks et al., 2017; Shapiro-Mendoza et al., 2009). Factors that affect SUID risk are often not independent of one another. Although the reasons for racial disparities are still incompletely understood, they are most likely due to a combination of variations in genetic susceptibility, socioeconomic status, and sleeping risk factors among different populations (Parks et al., 2017; El-Sayed et al., 2015). For instance, it has been reported that African-American infants are more likely to be placed to sleep in a non-supine position than white and Hispanic infants (Parks et al., 2017; Mathews et al., 2015).

The geographical distribution of infant deaths within Cook County also indicates populations at increased risk. Notably, 28% of the deaths in Cook County were clustered within only six zip codes, and more than half of the deaths in Cook County occurred in the city of Chicago. This finding is not surprising. Health outcomes vary widely throughout Cook County and within the city of Chicago, with clear and identifiable trends associated with race and socioeconomic status. This is borne out by disparities in average life expectancy within Cook County, which ranges from 61.7 years to 95.0 years depending on census tract area. Our data confirm that these disparities hold true for SUID within the county and contribute additional information for identifying neighborhoods for targeting interventions. The locations with the highest number of unexpected infant death are predominantly African-American communities that also have high rates of poverty (Place Matters for Health in Cook County, 2012).

We identified co-sleeping as a potentially avoidable risk factor. Colson et al. found that 13.5% of mothers of children less than 7 months of age reported that their infants usually co-slept. In 2010, this figure was 9.1% for white infants, 38.7% for African-American infants, and 20.5% for Hispanic infants (Colson et al., 2013). Comparatively, 48.3% of the infants in our dataset were co-sleeping. Previous studies have shown that caregivers sleep with their infants for multiple reasons, such as lack of an alternative location, convenience, desire to be close to their infants, and conflicting safety information from the media and family members (Eisenberg et al., 2015; Joyner et al., 2009; Joyner et al., 2010; Hirsch et al., 2018). Our findings may serve as baseline information in the future when evaluating the utility of interventional programs, such as the distribution of bassinettes, to prevent sleep-related infant deaths in Cook County.

It is important to note that the presence of sleep-related risk factors in unexplained infant deaths does not exclude the possibility that genetic factors also increase susceptibility to SUID. While no single genetic mutation has been linked to SUID, several rare gene variants, such as those of voltage-gated sodium channel subunits SCN4A and SCN5A, have been proposed as risk factors (Männikkö et al., 2018; Van Niekerk et al., 2017; Neubauer et al., 2017). In fact, Haas et al. describe identifying variants of genes associated with cardiovascular and metabolic diseases in 20% of unexpected infant deaths (Neubauer et al., 2017). A complex interplay of environmental and genetic factors contributes to the vulnerability of infants to sudden death, a concept originally described by Filiano and Kinney as the triple risk model (Filiano & Kinney, 1994). Our study contributes salient information regarding the prevalence of modifiable risk factors among infants who died unexpectedly during sleep.

There are several limitations to this study. The identification of sleep-related risk factors among the undetermined deaths depended on extracting descriptive information. Thus, it is possible that the presence of sleep-related risk factors was under-identified. This and the relatively small sample size in this study may have contributed to the absence of statistically significant differences. Additionally, the retrospective nature of the study, inconsistency in reporting, and the lack of a national standardized approach in the United States to survey these cases are all factors that hinder detailed characterizations of deaths. For instance, there is evidence that the decline in reported SIDS deaths since 2000 has been partially due to changes in reporting by coroners and medical examiners. Instead of attributing these deaths to SIDS, they have begun using other categories, such as “accidental suffocation and strangulation in bed” or “unknown” (Sudden Unexpected Infant Death and Sudden Infant Death syndrome: CDC/NCHS, 1990–2016; Quinlan et al., 2018; Shapiro-Mendoza et al., 2010; Shapiro-Mendoza et al., 2006). In Illinois, the lack of consistent reporting methods has been compounded by the fact that prior to July 2015, there were no mandatory child fatality reviews on sleep-related deaths in Illinois. In fact, Wu et al. showed that in Cook County, scene investigations of child deaths of undetermined intent were conducted in only 18.8% of cases between the years of 2005 and 2010 (Wu et al., 2017). Another limitation is the lack of relevant details, such as family member cigarette use, family housing stability, involvement with the Department of Child and Family Services (DCFS), whether other children in the family had died unexpectedly, and whether the family received Illinois Special Supplemental Nutrition Program for Women, Infants, and Children (WIC) or Medicaid support. Detailed medical histories of the infants; such as prenatal care, birth complications, and Apgar scores; were also not available. To address this lack of data, the CDC’s Division of Reproductive Health has created a new SUID monitoring system which combines information available in the VDRS with information from child death review teams, such as the infant’s medical records, and autopsy reports. At the time of the data analysis, this system was funded in only 16 states throughout the US and did not include Illinois (Shapiro-Mendoza et al., 2012; Sudden Unexpected Infant Death, 2018). Beginning in January 2019, Cook County data will begin to be included within the national SUID registry (SUID and SDY Case Registry: U.S, 2018). The SUID database will be an enhanced method for thorough and comprehensive surveillance of sleep-related infant deaths.

### Conclusions

The majority of sudden unexplained infant deaths in Cook County involved the presence of sleeping risk factors, suggesting that some of these deaths may be preventable. Several geographical areas within Chicago appear to be at increased risk, as are African American infants and infants under 2 months of age. Our results provide baseline data for future studies that may lead to targeted public health interventions in the effort to reduce infant mortality in Cook County.

## Abbreviations

AAP: American Academy of Pediatrics
ICD-10: International Classification of Disease, tenth revision
IDPH: Illinois Department of Public Health
IVDRS: Illinois Violent Death Reporting System
NVDRS: National Violent Death Reporting System
SUID: Sudden unexplained infant death
